# Tuberous Sclerosis Complex (Bourneville‐Pringle Disease) With Congenitally Right Solitary Kidney in a 14‐Year‐Old Male Adolescent

**DOI:** 10.1002/ccr3.73255

**Published:** 2026-07-28

**Authors:** Animaw Lingerew Dagnaw, Asressie Mamo Alemayehu, Gebre Hailu Dejen, Astarkew Alene Abejehu, Kindye Wale Kassie

**Affiliations:** ^1^ Department of Pediatrics and Child Health, School of Medicine, College of Medicine and Health Sciences Woldia University Ethiopia; ^2^ Department of Dermatovenereology, School of Medicine, College of Medicine and Health Sciences Woldia University Woldia Ethiopia; ^3^ Department of Radiology, School of Medicine, College of Medicine and Health Sciences Woldia University Woldia Ethiopia; ^4^ Department of Internal Medicine, School of Medicine, College of Medicine and Health Sciences Woldia University Woldia Ethiopia; ^5^ Department of Ophthalmology, School of Medicine, College of Medicine and Health Sciences Woldia University Woldia Ethiopia

**Keywords:** angiomyolipomas, autosomal dominant, bourneville‐pringle disease, congenital solitary kidney, tuberous sclerosis complex (TSC)

## Abstract

The coexistence of tuberous sclerosis complex (TSC) with a congenital solitary kidney is exceptionally rare and worsens prognosis. This combination increases the risk of renal failure, requiring meticulous monitoring, tailored surveillance, and renal preservation strategies. Early recognition, multidisciplinary care, and individualized management are essential to optimize outcomes in complex clinical scenarios.

**Abbreviations**
AML: angiomyolipoma
RCC: renal cell carcinoma
TSC: tuberous sclerosis complex

## 1 Introduction

Tuberous sclerosis complex (TSC) is a rare autosomal dominant genetic disease that is caused by mutation of the TSC1 or TSC2 gene. It is characterized by the formation of benign tumors and hamartomas in various organs of the body such as skin, brain, kidneys, and heart. TSC occurs at a rate of approximately 1 in 5000 to 10,000 live births. Kidneys are among the most commonly affected organs in patients with TSC, with angiomyolipomas (AMLs) and cysts being the most frequent findings. In children with TSC, solitary kidneys present a particularly hazardous situation, requiring special attention to prevent complications such as kidney failure. The most common observations in TSC are skin‐related, which are regarded as preliminary signs for the diagnosis in the majority of instances. Moreover, about 85% of patients with TSC experience epilepsy, which appears within the first year of life. Children with epilepsy frequently have cortical tubers known as glioneuronal hamartomas, subependymal nodules, and giant cell astrocytomas located in the subependymal region. Nonetheless, hamartomas are not restricted to the brain since they can also influence the heart and the kidneys as well. Additionally, no fewer than 50% of patients face cognitive challenges and cognitive impairment [1, 3].

## 2 Case Presentation

A 14‐year‐old male adolescent of black African descent presented to the dermatology outpatient department with facial skin lesions. The parents first observed the facial lesions when the boy was only three years old. The father recalls that when his son was 4 years old, he started having abnormal body movements that were characterized by sudden, brief jerks involving both arms and legs, consistent with generalized tonic‐clonic movements. These episodes occurred one or two times per week, each lasting only a few seconds, and continued for approximately three to four months. The frequency gradually decreased over time, and the episodes resolved completely without recurrence. Otherwise, he has no history of flank pain or urine color change or change in cognition as compared to his siblings or his peers. He was born at term to non‐consanguineous parents, and the labor and delivery process was uneventful. The family history was also not contributory.

On physical examination, he is well‐looking and vital signs are stable with oxygen saturation of 96%. On cutaneous examination, there are multiple skin‐colored to hyperpigmented papules and plaques on the face, especially over the cheek, nose, and forehead, consistent with angiofibromas as shown below in (Figure 1). Rest of the systemic examination including ophthalmic evaluation did not show any positive findings.

**FIGURE 1:**
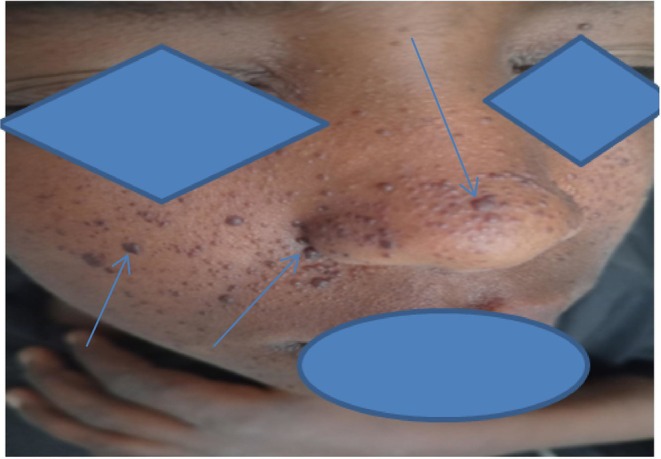
Multiple skin‐colored to hyperpigmented papules and plaques on the face, mainly over the cheek, nose and forehead, consistent with angiofibroma.

## 3 Methods

### 3.1 Investigations

With provisional diagnosis of tuberous sclerosis complex, we conducted laboratory tests, such as routine blood work, liver and kidney function assessments, urine analyses; the results of all these tests were normal as mentioned in the Table 1 below. The ECG shows normal sinus rhythm and echocardiographic evaluation shows no cardiac mass. Abdominal imaging (ultrasound and CT renal protocol) revealed multiple right renal angiomyolipomas, small cortical cysts, and absence of the left kidney, consistent with congenital solitary kidney (Figures 2 and 3). Brain MRI demonstrated bilateral subependymal nodules along the lateral ventricles with associated cortical and subcortical tubers involving the right parietal, temporal and bilateral occipital cortex (Figures 4 and 5). Axial sagittal and coronal CT scan had also features of multiple sclerotic bone lesions in the vertebral body and pelvic bone (Figure 6).

**TABLE 1:** Laboratory investigation results.

CBC	WBC‐5000 mm^3^, Hemoglobin‐12 g/dL, hematocrite‐37%, platelet −150,000/L
Renal function test (mg/dl)	Creatinin‐0.5
Urine analysis	Albumin–negative No WBC or RBC
LFT (U/L)	ALP‐150, ALT−35, AST‐30

**FIGURE 2:**
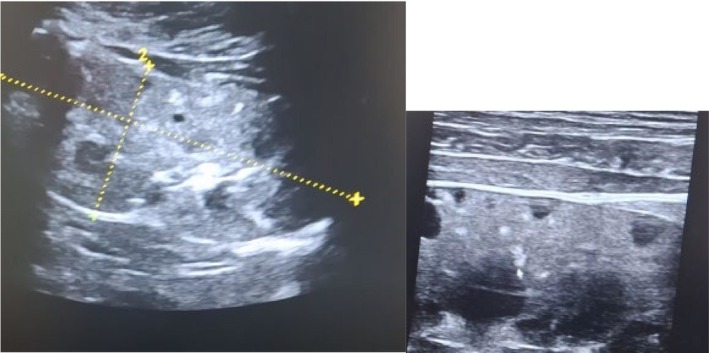
Curvilinear and linear probe ultrasound images showing multiple tiny echogenic nodules and cystic lesions throughout the right renal cortex consistent with multiple angiomyolipoma and multiple renal cysts in the TSC. Left renal fossa (not shown here) was empty and no other abdominal findings were noted.

**FIGURE 3:**
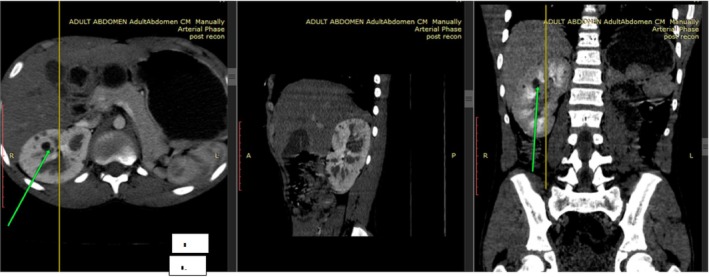
Post‐contrast CT scan images multiple subcentimetric fat‐containing renal lesion throughout the right renal cortex/indicated by green arrows consistent with multiple angiomyolipoma. Multiple small renal cortical cystic lesions were seen as well. The left renal fossa is empty and no renal tissue was seen in any ectopic location.

**FIGURE 4:**
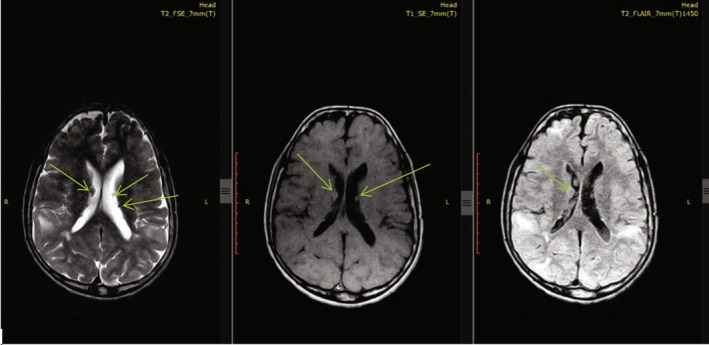
Brain MRI showing multiple small‐sized sub ependymal nodules in the lateral ventricular walls bilaterally that display hypo intense signal on T2 and FLAIR and iso to hyper intense signal on T2W consistent with sub ependymal tubers.

**FIGURE 5:**
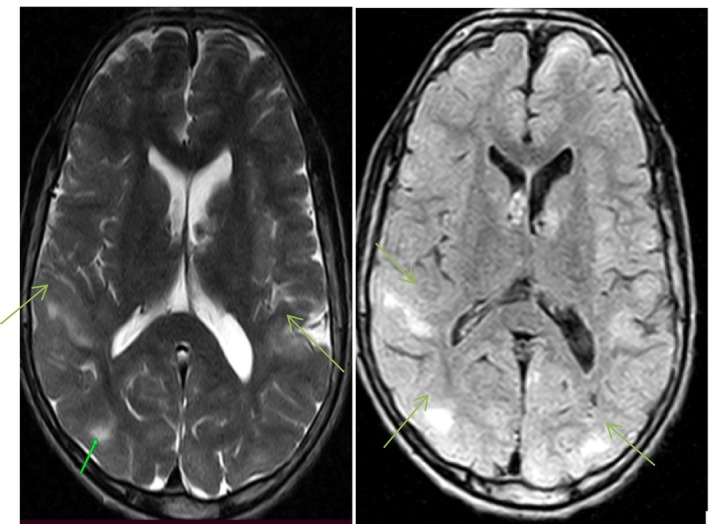
Brain MRI shows multiple cortical and subcortical areas of altered signal intensity displaying bright signal on T2/FLAIR denoting cortical/subcortical tubers involving the right parietal, temporal and bilateral occipital cortex.

**FIGURE 6:**
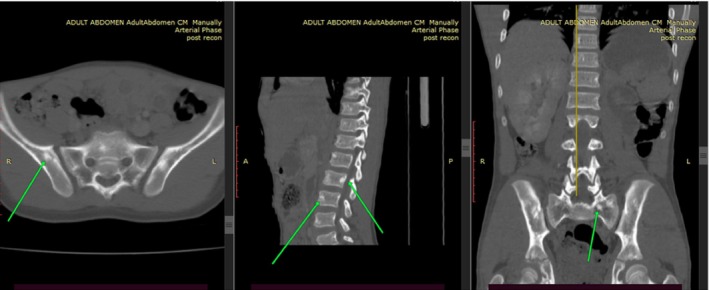
Axial, sagittal, and coronal CT scan showing multiple sclerotic bone lesions in the vertebral body and pelvic bone.

### 3.2 Management and Follow‐Up

The patient was diagnosed with definite TSC based on major criteria, including facial angiofibromas, cortical and subcortical tubers, subependymal nodules, renal angiomyolipomas, renal cysts, and sclerotic bone lesions. Although seizures occurred in early childhood, they resolved spontaneously without medical intervention, and no current neurological deficits are present.

Renal function tests remained within normal limits despite the absence of the left kidney and the presence of multiple lesions in the right kidney. Cardiac and ophthalmic evaluations were unremarkable, and no cognitive impairment was noted. The patient was clinically stable and asymptomatic at the time of diagnosis.

Given the multisystem involvement and the potential for progressive complications, a multidisciplinary follow‐up plan was initiated. Renal surveillance included regular abdominal imaging (ultrasound or MRI) every 6–12 months to monitor the size and number of angiomyolipomas and cysts, with attention to potential hemorrhagic risk or renal function decline. Neurological monitoring involved periodic neurological assessments and brain imaging to evaluate for progression of cortical tubers or development of subependymal giant cell astrocytomas (SEGAs). Dermatological care consisted of ongoing dermatology follow‐up for management of facial angiofibromas, including consideration of topical or laser therapy if lesions become symptomatic or cosmetically concerning. Genetic counseling was offered to the family to discuss the hereditary nature of TSC and implications for future offspring. Our patient is only on supportive treatment as he is clinically stable. Psychosocial support was provided to address any emotional or social challenges related to the diagnosis and its manifestations.

The patient and family were educated about the chronic nature of TSC, the importance of routine surveillance, and signs of potential complications that warrant immediate medical attention. At the most recent follow‐up, the patient remained clinically stable with no new symptoms, and renal function continued to be preserved.

## 4 Discussion

Tuberous sclerosis, often known as bourneville‐Pringle disease, is a multisystem neurocutaneous disorder that affects individuals of all ages, including both children and adults. It features the formation of hamartomatous lesions in different organs, such as the brain, kidneys, lungs, heart, skin, and skeletal system. The integumentary system is the most frequently impacted, involved in 81%–95% of cases of tuberous sclerosis. The most common skin lesions consist of hypomelanotic macules, fibrous cephalic plaques, angiofibromas mainly affecting the malar regions of the face, and ungual fibromas [1].

Renal involvement in TSC is prevalent, with angiomyolipomas (AMLs) and cysts representing the most commonly seen abnormalities, and their occurrence rises with age. Renal AML, a noncancerous hamartoma, may arise sporadically or be linked to tuberous sclerosis complex (TSC). However, a congenital solitary kidney is seldom linked to tuberous sclerosis, and to the best of our knowledge, there exists only one documented case report in the literature. When a patient possesses a solitary kidney, the handling of kidney issues becomes even more crucial because of the heightened risk of kidney failure. A concerning complication of AMLs is their progressive growth, which can lead to bleeding within the lesion, causing pain and impairing kidney function. The likelihood of bleeding rises with size. Moreover, individuals with tuberous sclerosis and renal lesions may experience renin‐dependent hypertension and are prone to chronic kidney disease from the replacement and compression of renal parenchyma, necessitating consistent blood pressure monitoring. The existence of symptoms and the size of the renal lesion (typically > 4 cm) necessitates medical intervention, primarily with mTOR inhibitors to diminish or halt the growth of these lesions. Conservative surgery is often required because of the numerous tumors and the bilateral nature of the renal lesions [2, 3].

Post‐contrast CT scan images of the kidney (Figure 3) reveal numerous subcentimetric fat‐containing renal lesions across the right renal cortex, marked by green arrows, consistent with multiple angiomyolipomas. Additionally, several small renal cortical cystic lesions are also observed. The left renal fossa is void, and no renal tissue was observed in any ectopic sites. The presence of fat in the lesions needs verification through appropriate imaging techniques, as renal cell carcinoma (RCC) and AML can exhibit similar acoustic characteristics. CT is frequently utilized to assess the cases with the highest likelihood of being AML. When lesions do not show apparent fat content, advanced imaging methods like MRI are useful for verifying the diagnosis of AML [3, 4, 5]. In our patient, the post‐contrast CT scan of the abdomen showed typical features of several fat‐containing lesions distributed across the right kidney.

The fact that the patient has subcentimetric size of renal lesions and the patient is asymptomatic with normal urine analysis and renal function requires meticulous regular follow up with no medical intervention. Unfortunately, surgical interventions are challenging due to the presence of only a solitary kidney of our particular patient.

Another characteristic of the TSC is neurologic involvement. Cortical and subcortical nodules, sub ependymal nodules (SENs), sub ependymal giant cell astrocytoma (SEGA), associated with seizures, intellectual impairment, and autism spectrum disorder (ASD) are among the prevalent appearances of TSC. Infantile spasm is the most common presentation of seizure disorders in up to 85% of cases with this neurologic disease in tuberous sclerosis, particularly in newborns or young children. Other self‐limited seizure syndromes within the TSC spectrum are also possible. Neuropsychiatric conditions linked to tuberous sclerosis, such as attention deficit hyperactivity disorder and autism spectrum disorder [6, 7].

Our patient brain MRI showed multiple small‐sized sub ependymal nodules in the lateral ventricular walls bilaterally that display hypo intense signal on T2 and FLAIR and iso to hyper intense signal on T2W consistent with sub ependymal nodule. Multiple cortical and subcortical areas of altered signal intensity displaying bright signal on T2/FLAIR denoting cortical/subcortical tubers are also suggest typical features of tuberous sclerosis. Early detection and early initiation of antiepileptic drugs and mTOR inhibitors are the mainstay of treatment depending on the presence of symptoms and size of the lesions. Clinically, the patient has a history of seizure during his early childhood period which gets improved spontaneously and he is not taking any medication [8, 9].

Sclerotic bone lesions are among the skeletal manifestations of TSC that are mostly detected incidentally. Sclerotic bone lesions can present in about 40% to 50% of patients and are currently incorporated as minor clinical criteria in the diagnostic criteria by the Updated international tuberous sclerosis consensus group [9].

The diagnosis of bourneville disease is primarily based on clinical findings with the aid of radiological imaging modalities, following criteria of major and minor findings. According to the international tuberous sclerosis consensus group, the clinical diagnostic criteria consist of 12 major and 7 minor criteria. For a definitive diagnosis of tuberous sclerosis complex, either 2 major or 1 minor and 2 minor clinical manifestations are required. Hence, our case having angiofibromatous skin lesions, brain cortical and sub ependymal nodules, sclerotic bone lesions, renal angiomyolipoma fulfills the diagnostic criteria. The presence of a solitary kidney, in addition to the typical renal cyst or renal angiomyolipoma, makes the management more difficult and challenging [8, 10].

This case highlights the multisystem involvement characteristic of TSC, including dermatological, neurological, renal, and skeletal manifestations. While these features are well described in the literature, the rarity of a solitary kidney in TSC makes this case particularly instructive. In such patients, conventional nephron‐sparing surgery carries unacceptable risk, as any loss of renal parenchyma could precipitate renal failure. Similarly, embolization—though effective in controlling hemorrhage—assumes heightened stakes when performed on the only kidney. These limitations underscore the critical role of mTOR inhibitors as a prophylactic and therapeutic strategy, offering a noninvasive means to reduce AML burden and preserve renal function. Equally imperative is the avoidance of nephrotoxic agents, given the absolute dependence on solitary kidney [11].

Thus, while the broader spectrum of TSC complications provides context, the unique contribution of this case lies in emphasizing a multidisciplinary, kidney‐preserving approach to AML management in solitary kidneys. This focus not only informs clinical decision‐making but also reinforces the importance of individualized care strategies in rare TSC presentations.

## 5 Conclusion

The coexistence of TSC with a solitary kidney presents a unique scenario that significantly impacts management strategies and long‐term prognosis. While renal involvement is common in TSC (angiomyolipomas, cystic disease), the presence of a solitary kidney further complicates management. Since angiomyolipomas (AMLs) can cause bleeding or progressive loss of renal function, a solitary kidney poses higher risks for end‐stage renal disease (ESRD). Standard interventions (e.g., mTOR inhibitors like everolimus) must be tailored to minimize renal toxicity in a patient with only one functioning kidney. Any renal embolization or surgical intervention carries higher stakes, as the patient lacks a second compensatory kidney. With only one functional kidney, the patient has an increased risk of renal insufficiency or ESRD, affecting lifespan and quality of life. So such patients require closer nephrology surveillance, periodic GFR assessments, and kidney function monitoring. Certain medications (e.g., nephrotoxic antibiotics or NSAIDs) must be strictly avoided to prevent further renal damage.

We presented this case report due to its rare occurrence of tuberous sclerosis with solitary kidney, unique management challenges, and altered prognosis. Understanding such cases guides clinical decision‐making, ensuring optimal renal preservation strategies.

## Author Contributions

**Animaw Lingerew Dagnaw:** conceptualization, investigation, methodology, writing – original draft, writing – review and editing. **Asressie Mamo Alemayehu:** conceptualization, investigation, writing – original draft. **Gebre Hailu Dejen:** conceptualization, investigation, methodology. **Astarkew Alene Abejehu:** conceptualization, investigation, methodology. **Kindye Wale Kassie:** conceptualization, investigation, methodology.

## Funding

The authors have nothing to report.

## Disclosure

The images were taken by the authors and we confirm that they are original with no duplication and have not been previously published in whole or in part. The authors also declare that AI‐assisted tools were not used in the writing of this manuscript.

## Ethics Statement

The authors have nothing to report.

## Consent

Written informed consent was taken from the child's parent to take pictures and the clinical information to be published for academic purpose anonymously. A copy of the written consent is available for review by the editor‐in‐Chief of this journal.

## Conflicts of Interest

The authors declare no conflicts of interest.

## Data Availability

The data that support the findings of this study are available from the corresponding author upon reasonable request.
